# Supplementary material for: Investigation of the associations between physical activity, self-regulation and educational outcomes in childhood
Source: PLoS One. 2021 May 19;16(5):e0250984. doi: 10.1371/journal.pone.0250984 (PMC8133416; doi:10.1371/journal.pone.0250984)
Supplement: S1 Table — (DOCX) [file pone.0250984.s001.docx]

**S1 Table.** Sample description and comparison of age participants.

|  | Age 7 | | | Age 11 | | | Age 14 | | |
| --- | --- | --- | --- | --- | --- | --- | --- | --- | --- |
|  | *N* | *M* | *SD* | *N* | *M* | *SD* | *N* | *M* | *SD* |
| **Emotional Regulation** |  |  |  |  |  |  |  |  |  |
| Item 1 (ER 1) | 3974 | 1.60 | 0.75 |  |  |  |  |  |  |
| Item 2 (ER 2) | 3974 | 1.90 | 0.76 |  |  |  |  |  |  |
| Item 3 (ER 3) | 3974 | 1.78 | 0.73 |  |  |  |  |  |  |
| Item 4 (ER 4) | 3973 | 1.67 | 0.74 |  |  |  |  |  |  |
| Item 5 (ER 5) | 1836 | 1.30 | 0.59 | 2935 | 1.19 | 0.67 | 2212 | 1.42 | 0.65 |
| Item 6 (ER 6) | 2716 | 1.55 | 0.38 | 2935 | 1.43 | 0.78 | 2212 | 1.61 | 0.69 |
| **Behavioural Regulation** |  |  |  |  |  |  |  |  |  |
| Item 1 (BR1) | 3973 | 2.27 | 0.66 |  |  |  |  |  |  |
| Item 2 (BR2) | 3973 | 2.75 | 0.46 |  |  |  |  |  |  |
| Item 3 (BR 3) | 2709 | 2.34 | 0.71 | 2935 | 2.46 | 0.66 | 2212 | 2.36 | 0.64 |
| Item 4 (BR 4) | 2710 | 2.32 | 0.63 | 2935 | 2.42 | 0.77 | 2209 | 2.23 | 0.62 |
| Item 5 (BR 5) | 2714 | 1.11 | 0.38 | 2935 | 1.09 | 0.57 | 2208 | 1.51 | 0.68 |
| **Physical Activity** |  |  |  |  |  |  |  |  |  |
| Item 1 (Objective) (PA1) | 4043 | 62.78 | 22.26 |  |  |  | 1557 | 128.01 | 72.65 |
| Item 2 (Subjective) (PA2) | 3874 | 1.35 | 0.56 |  |  |  |  |  |  |
| Item 3 (Subjective) (PA3) | 3871 | 1.66 | 0.68 |  |  |  |  |  |  |
| Item 4 (Objective) (PA4) |  |  |  |  |  |  | 3500 | 2.75 | 1.10 |
| Item 5 (Subjective): |  |  |  |  |  |  |  |  |  |
| *Cycling* (PA5a) |  |  |  |  |  |  | 1005 | 10.96 | 46.00 |
| *Individual ball games and training* (PA5b) | |  |  |  |  |  | 1005 | 13.26 | 42.81 |
| *Jogging, running, walking, hikin*g (PA5c) | |  |  |  |  |  | 1005 | 41.02 | 80.30 |
| *Team ball games and training* (PA5d) | |  |  |  |  |  | 1005 | 39.78 | 94.85 |
| *Swimming and other water sports* (PA5e) | |  |  |  |  |  | 1005 | 17.12 | 62.47 |
| *Other exercise and sports (dancing, skiing, gymnastics)* (PA5f) | | | |  |  |  | 1005 | 52.43 | 105.32 |
| **Academic Achievement** |  |  |  |  |  |  |  |  |  |
| *Standardised Test* |  |  |  |  |  |  |  |  |  |
| Math | 4034 | 100.29 | 15.16 |  |  |  |  |  |  |
| Literacy | 4031 | 115.59 | 16.78 |  |  |  |  |  |  |
| *Teacher Report* |  |  |  |  |  |  |  |  |  |
| English | 2689 | 2.49 | 1.00 | 2936 | 2.46 | 0.97 |  |  |  |
| Math | 2681 | 2.58 | 0.94 | 2930 | 2.39 | 0.98 |  |  |  |
| Science | 2691 | 2.61 | 0.79 | 2923 | 2.45 | 0.83 |  |  |  |
| IT Technology | 2689 | 2.74 | 0.66 | 2925 | 2.51 | 0.70 |  |  |  |
| Arts and Design | 2692 | 2.74 | 0.70 | 2914 | 2.67 | 0.76 |  |  |  |
| Music |  |  |  | 2884 | 2.75 | 0.72 |  |  |  |
| Physical Education | 2688 | 2.68 | 0.69 | 2930 | 2.62 | 0.78 |  |  |  |
|  |  |  |  |  |  |  |  |  |  |

SD, standard deviation; BR, behavioural regulation; ER, emotional regulation; PA, physical activity.
