# Supplementary material for: Investigation of the associations between physical activity, self-regulation and educational outcomes in childhood
Source: PLoS One. 2021 May 19;16(5):e0250984. doi: 10.1371/journal.pone.0250984 (PMC8133416; doi:10.1371/journal.pone.0250984)
Supplement: S2 Table — (DOCX) [file pone.0250984.s002.docx]

**S2 Table.** Correlations Within and Between Physical Activity, Emotional Regulation, Behavioural Regulation, Index of Multiple Risk

|  | 1 | 2 | 3 | 4 | 5 | 6 | 7 | 8 |
| --- | --- | --- | --- | --- | --- | --- | --- | --- |
|  |  |  |  |  |  |  |  |  |
| 1. Emotional Regulation (age 7) |  |  |  |  |  |  |  |  |
| 2. Emotional Regulation (age 11) | 0.30 |  |  |  |  |  |  |  |
| 3. Emotional Regulation (age 14) | 0.24 | 0.24 |  |  |  |  |  |  |
| 4. Behavioural Regulation (age 7) | 1.00 | -0.30 | -0.22 |  |  |  |  |  |
| 5. Behavioural Regulation (age 11) | -0.29 | -0.99 | -0.24 | 0.31 |  |  |  |  |
| 6. Behavioural Regulation (age 14) | -0.02 | -0.03 | -0.19 | 0.02 | 0.04 |  |  |  |
| 7. Physical Activity (age 7) | 0.67 | 0.68 | 0.05 | -0.54 | -0.57 | 0.00 |  |  |
| 8. Physical Activity (age 14) | 0.12 | 0.12 | 0.31 | -0.09 | -0.10 | 0.04 | 0.17 |  |
| 9. Index of Multiple Risk (age 5) | -0.21 | -0.26 | -0.30 | 0.20 | 0.24 | 0.07 | -0.52 | 0.20 |
|  |  |  |  |  |  |  |  |  |
