# Supplementary material for: Investigation of the associations between physical activity, self-regulation and educational outcomes in childhood
Source: PLoS One. 2021 May 19;16(5):e0250984. doi: 10.1371/journal.pone.0250984 (PMC8133416; doi:10.1371/journal.pone.0250984)
Supplement: S3 Table — (DOCX) [file pone.0250984.s003.docx]

**S3 Table.** Correlations Within and Between Physical Activity, Emotional Regulation, Behavioural Regulation, Index of Multiple Risk and Academic Achievement

|  | 1 | 2 | 3 | 4 | 5 | 6 | 7 | 8 |
| --- | --- | --- | --- | --- | --- | --- | --- | --- |
|  |  |  |  |  |  |  |  |  |
| 1. Emotional Regulation (age 7) |  |  |  |  |  |  |  |  |
| 2. Emotional Regulation (age 11) | 0.76 |  |  |  |  |  |  |  |
| 3. Behavioural Regulation (age 7) | -0.80 | -0.81 |  |  |  |  |  |  |
| 4. Behavioural Regulation (age 11) | -0.80 | -0.81 | 0.86 |  |  |  |  |  |
| 5. Physical Activity (age 7) | 0.86 | 0.88 | -0.93 | -0.92 |  |  |  |  |
| 6. Index of Multiple Risk (age 5) | -0.30 | -0.30 | 0.32 | 0.32 | -0.34 |  |  |  |
| 7. Prior Academic Achievement & Cognitive Ability (age 5) | 0.50 | 0.51 | -0.54 | -0.54 | 0.59 | -0.92 |  |  |
| 8. Academic Achievement (age 7) | 0.77 | 0.51 | -0.67 | -0.54 | 0.58 | -0.17 | 0.33 |  |
| 9. Academic Achievement (age 11) | 0.63 | 0.77 | -0.68 | -0.81 | 0.73 | -0.36 | 0.5 | 0.33 |
|  |  |  |  |  |  |  |  |  |
