# Supplementary material for: Investigation of the associations between physical activity, self-regulation and educational outcomes in childhood
Source: PLoS One. 2021 May 19;16(5):e0250984. doi: 10.1371/journal.pone.0250984 (PMC8133416; doi:10.1371/journal.pone.0250984)
Supplement: S4 Table — (DOCX) [file pone.0250984.s004.docx]

**S4 Table**. Fit statistics for longitudinal measurement invariance

|  | χ2 (df) | *p* | RMSEA (90% CI) | CFI | TLI | SRMR |
| --- | --- | --- | --- | --- | --- | --- |
| **Model** |  |  |  |  |  |  |
| Configural | 2996.50 (300) | *< .001* | .05 (.05 - .05) | .99 | .99 | .04 |
|  |  |  |  |  |  |  |
| Strong | 47359.83 (350) | *< .001* | .19 (.19 - .19) | .98 | .98 | .04 |
|  |  |  |  |  |  |  |
| Strict | 49270.38 (367) | *< .001* | .19 (.19 - .19) | .98 | .98 | .04 |
|  |  |  |  |  |  |  |

All fit indices based WLSMV estimator.

df, degrees of freedom; RMSEA, root mean square error of approximation; CI, confidence interval; CFI, comparative fit index; TLI, tucker–lewis index; SRMR standardised root mean square residual; WLSMV, weighted least squares mean and variance.
