# Supplementary figures and images for: Investigation of the associations between physical activity, self-regulation and educational outcomes in childhood
Source: PLoS One. 2021 May 19;16(5):e0250984. doi: 10.1371/journal.pone.0250984 (PMC8133416; doi:10.1371/journal.pone.0250984)

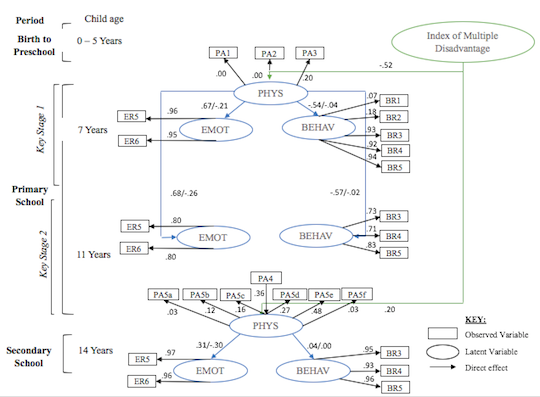

Supplement: S1 Fig — PA, physical activity; ER, emotional regulation; BR, behavioural regulation; PHYS, physical activity; EMOT, emotional regulation; BEHAV, behavioural regulation. The parameters on the left side of the slash are without controlling for SES, on the right controlled for SES. (TIF) [file pone.0250984.s005.tif]

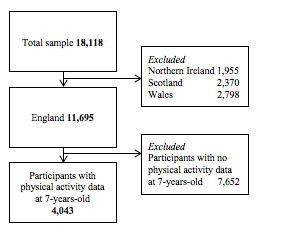

Supplement: S2 Fig — Inclusion criteria for sub-sample from the Millennium Cohort Study London: Centre for Longitudinal Studies, Institute of Education, University of London. Maximum number of participants in sample. (TIF) [file pone.0250984.s006.tif]
